# Supplementary material for: Epigenetic regulation of nuclear lamina-associated heterochromatin by HAT1 and the acetylation of newly synthesized histones
Source: Nucleic Acids Res. 2021 Nov 12;49(21):12136–51. doi: 10.1093/nar/gkab1044 (PMC8643632; doi:10.1093/nar/gkab1044)
Supplement: gkab1044_Supplemental_Files [file gkab1044_supplemental_files.zip › Popova_et_al_2021__Revision_Supplementary_Material-.pdf]

Supplementary Information

Supplementary Methods

APEX2 Construct Localization

To evaluate localization of the constructs, cell fractionations were performed. First, cells were transfected as described in the Materials and Methods section of this paper. Cells were collected, washed with PBS, and resuspended in Lysis Buffer 1 (50mM Tris-HCl, pH 7.5, 20mM NaCl, 1mM EDTA, 10% glycerol, 0.5% NP-40, and 0.25% TritonX-100 supplemented with 1X Proteinase inhibitor cocktail (Roche), 2mM sodium vanadate, 10mM sodium butyrate, and 1mM DTT), incubated for 15min at 4°C with rotation and centrifuged at 400g for 5min at 4°C. Supernatant was collected as the cytosolic fraction. Next, the samples were washed in Lysis buffer 2 (50mM Tris-HCl, pH 7.5, 20mM NaCl, 1mM EDTA, and 1mM EGTA supplemented with 1X Proteinase inhibitor cocktail (Roche), 2mM sodium vanadate, 10mM sodium butyrate, and 1mM DTT). Samples were incubated for 5 min at 4°C with rotation and centrifuged at 400g for 5min at 4°C. Supernatant was discarded and the cell pellets were resuspended in Lysis buffer 3 (50mM Tris-HCl, pH 7.5, 250mM NaCl, 1mM EDTA, 0.5mM EGTA, 0.1% sodium deoxycholate, and 0.5% N-laurylsarcosine, supplemented with 1X Proteinase inhibitor cocktail (Roche), 2mM sodium vanadate, 10mM sodium butyrate, and 1mM DTT). Samples were sonicated using Diagenode Bioruptor Sonicator on high setting for 10 min with 30sec on/off until lysates no longer appeared viscous. Samples were centrifuged at 20000g for 15min at 4°C, supernatant was collected as the nuclear fraction. Protein lysates were boiled in 2X Laemilli buffer, loaded on an SDS-PAGE gel,

transferred to a membrane using standard protocols and probed with anti-Hat1 antibody (Abcam, ab193097), anti-V5 antibody (Invitrogen, MA5-15253), anti-lamin B1 antibody and anti-GAPDH antibody (Abcam, ab9485) as loading controls.

Histone Sample Preparation for Epiprofile

Nuclei were isolated and histones extracted as follows. Briefly, 2 x 10^7 cells were harvested and fractionated into cytoplasmic proteins and nuclei with lysis buffer 1 (LB1 – 50 mM Tris-HCl, pH 7.5, 20 mM NaCl, 1 mM EDTA, 1% Glycerol (vol/vol), 0.5% NP-40 (vol/vol), 0.25% Triton X-100 (vol/vol), supplemented with 1x Roche cOmplete protease tablet, 2 mM Sodium Vanadate, 10 mM Sodium Butyrate, 1 mM DTT) incubated with rotation for 15 min at 4 °C and spun down at 400 *xg* for 5 min at 4 °C. Nuclei were washed with lysis buffer 2 (LB2 – 50 mM Tris-HCl, pH 7.5, 20 mM NaCl, 1 mM EDTA, 0.5 mM EGTA, supplemented with 1x Roche cOmplete protease tablet, 2 mM Sodium Vanadate, 10 mM Sodium Butyrate, 1 mM DTT), incubated and spun as before. Nuclear pellets were suspended in 0.4N H_2SO_4 and incubated for 2 h over ice with intermittent vortexing. Cellular debris was pelleted at >20000 *xg* for 15 min at 4 °C, supernatant transferred to 2 mL tube and histones precipitated by addition of ice-cold acetone and incubation O/N at -20 °C.

Samples were washed with ice-cold acetone twice prior to suspension in ABC (100 mM) and quantification via Bradford. Histones were prepared for bottom-up DIA as follows. Briefly, histone proteins (100 µg) were incubated for 15 min at RT with ACN:PA (3: 1 vol/vol) four times to ensure complete conversion of unmodified and mono-methylated lysine residues. Histones were digested with trypsin (1:25 wt/wt) 400 rpm at 37 °C O/N. Histone peptides were incubated with ACN:PA twice to convert all of the newly formed N-termini, dried in a vacuum concentrator and quantified via Nanodrop to estimate concentration and yield prior to mass spectrometry analysis.

Data-Independent Acquisition Tandem Mass Spectrometry (DIA-MS/MS)

Histone peptides (1200 ng) were separated with a PepMap C18 column (Thermo Fisher Scientific Cat No ES800A 3 μm, 100 Å, 75 μm x 15 cm) coupled to a Q-Exactive Plus Hybrid Quadrupole Orbitrap mass spectrometer (Thermo Fisher Scientific) by increasing 300 nL/min Buffer B (0.1% FA, ACN) over 75 min as follows: desalt/load peptides on trap 0-5 min 2% B, 5-45 min 45% B, 45-52 min 55% B, 52-56 min 95% B, 56-64 min hold at 95% B, equilibrate to 2% B for 64-75 min. Spectra were collected in data-independent acquisition (DIA) mode with the following parameters: MS1- resolution 35k, AGC target 3e6, maximum fill time 200 ms over a scan range 300-1100 *m/z,* MS2 – resolution 17.5k, AGC target 1e6 with a loop count of 8 and a sliding isolation window of 50 *m/z*. Raw data files were analyzed with EpiProfile 2.0 [194]. Significance was determined with Welch's t-test.

Immunofluorescence

Three HAT1 +/+ or HAT1 -/- MEF cell lines were seeded in equal quantities on coverslips and allowed to attach for 24 h. Cells were then permeabilized with 0.5% Triton X-100 and fixed with 4% PFA simultaneously for 15 min, rinsed with PBS, and fixed again with 4% PFA for 10 min at room temperature. After several PBS washes, cells were blocked with 5% BSA for 1 h at room temperature. BSA was removed with PBS washes and primary antibodies were diluted in 1% BSA, 0.3% Triton X-100 and added to cells overnight at 4 °C (Anti-LaminB1(ms), 1:200, Abcam cat. no: ab8982; Anti-LaminA/C(rb), 1:200, Abcam cat. no: ab133256). The following day, cells were incubated with secondary antibodies (Alexa Fluor 488-conjugated anti-rabbit, 1:250, Molecular Probes; Alexa Fluor 594-conjugated anti-mouse, 1:250, Molecular Probes) for 1 h at room temperature, antibody was removed with PBS, nuclei were stained with 20 mM Hoechst 33342 Fluorescent Stain and mounted on slides using Vectashield. Slides were analyzed under a Zeiss LSM 900 Airyscan 2 Point Scanning Confocal microscope. Images were acquired using

Zen Blue 3.0 and quantification was completed using ImageJ version 1.52t. Quantification consisted of identifying nuclear regions using Hoechst channel then measuring region area, intensity mean, and integrated density of 488 or 561 channel. Data was analyzed and plots generated using RStudio Version 1.2.5042 running R Version 4.0.0.

Data Availability

RAW mass spectrometry proteomics data reported in this paper has been deposited to PRIDE via massIVE and can be downloaded through the mass spectrometry interactive virtual environment (massIVe) FTP (ftp://massive.ucsd.edu/MSV000087497/) or the accession number (PXD026232) through PRIDE. Further, processed mzML, mztab and the search engine results file generated during the database search for this paper are also deposited with PXD026232.

Supplementary Figure Legends

Supplementary Figure 1. HAT1-dependent chromatin accessibility sites localize to heterochromatic regions of the genome. A. Pie chart displaying the distribution of HAT1-dependent sites of chromatin accessibility to the indicated genomic features. B. HAT1-dependendent sites of chromatin accessibility were localized to the indicated GC-content isochores. C. Locations of HAT1-dependent sites of chromatin accessibility, indicated as vertical lines are shown above a karyoplot of chromosome 1. Below the karyoplot is a graph of gene density on chromosome 1.

Supplementary Figure 2. Chromatin accessibility patterns are similar between primary and immortalized MEFs. A. Sliding window averages of log2 fold change of

ATAC-Seq data from primary and immortalized HAT1$^{+/+}$ and HAT1$^{-/-}$ MEFs. B. Genome browser view of an 8 Mb region of chromosome 6 showing ATAC-Seq data from primary and immortalized HAT1$^{+/+}$ and HAT1$^{-/-}$ MEFs.

Supplementary Figure 3. Certain histone modifications are enriched in absence of HAT1. A. The abundance of peptides containing the indicated modification on histone H3 (top) and histone H4 (bottom) in HAT1$^{+/+}$ and HAT1$^{-/-}$ MEFs (n=4 for each genotype) is plotted as relative abundance (left) or as enrichment/depletion in HAT1$^{-/-}$ cells. B. Histones were isolated from two independent HAT1+/+ and HAT1-/- MEF cell lines and Western blots were probed with the indicated antibodies. The difference in the abundance of H3 K9me2 and K9me3 was quantitated relative to total histone H3.

Supplementary Figure 4. Genomic distribution of sites of accessibility that increase in the absence of HAT1 relative to HAT1-depebndent RNA-Seq changes. Sites of increased chromatin accessibility in HAT1-/- MEFs are indicated above the karyoplot of each chromosome. Below each chromosome is indicated the location of differentially expressed RNAs.

Supplementary Figure 5. HADs colocalize with cLADs. Karyoplots of chromosome 1 and chromosome 16 are shown with the location of HADs above each chromosome and the location of constitutive LADs (cLADs) below.

Supplementary Figure 6. HAT1 does not effect lamin abundance or localization. A. Whole cell extracts from $HAT1^{+/+}$ and $HAT1^{-/-}$ MEFs were resolved by SDS-PAGE and analyzed by Western blotting with the indicated antibodies. B. $HAT1^{+/+}$ and $HAT1^{-/-}$ MEFs were were visualized with anti-lamin A/C antibodies and DAPI. The integrated density of lamin A/C signal was determined for cells of each genotype and plotted. C. $HAT1^{+/+}$ and $HAT1^{-/-}$ MEFs were were visualized with anti-lamin B antibodies and DAPI. The integrated density of lamin B signal was determined for cells of each genotype and plotted.

Supplementary Table 1. ATAC-Seq sites of differential accessibility in $HAT1^{+/+}$ and $HAT1^{-/-}$ MEFs.

Supplementary Table 2. Location of computationally derived HADs in pMEFs.

A

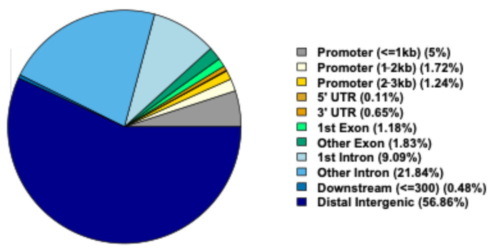

B

ISOCHORE	GC CONTENT	HAT1-DEPENDENT SITES (%)
L1	<37%	16
L2	37-41%	49
H1	41-46%	25
H2	46-53%	8
H3	>53%	0

C

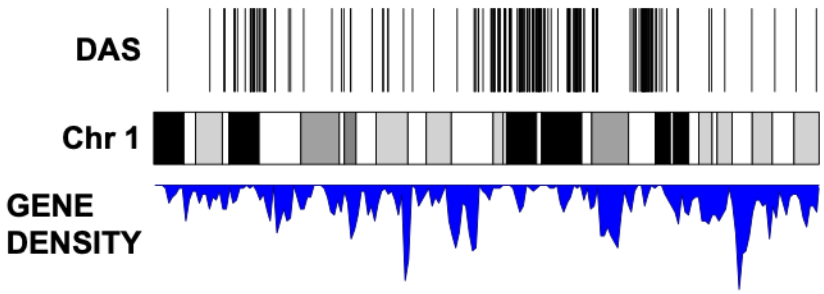

Supplementary Figure 1

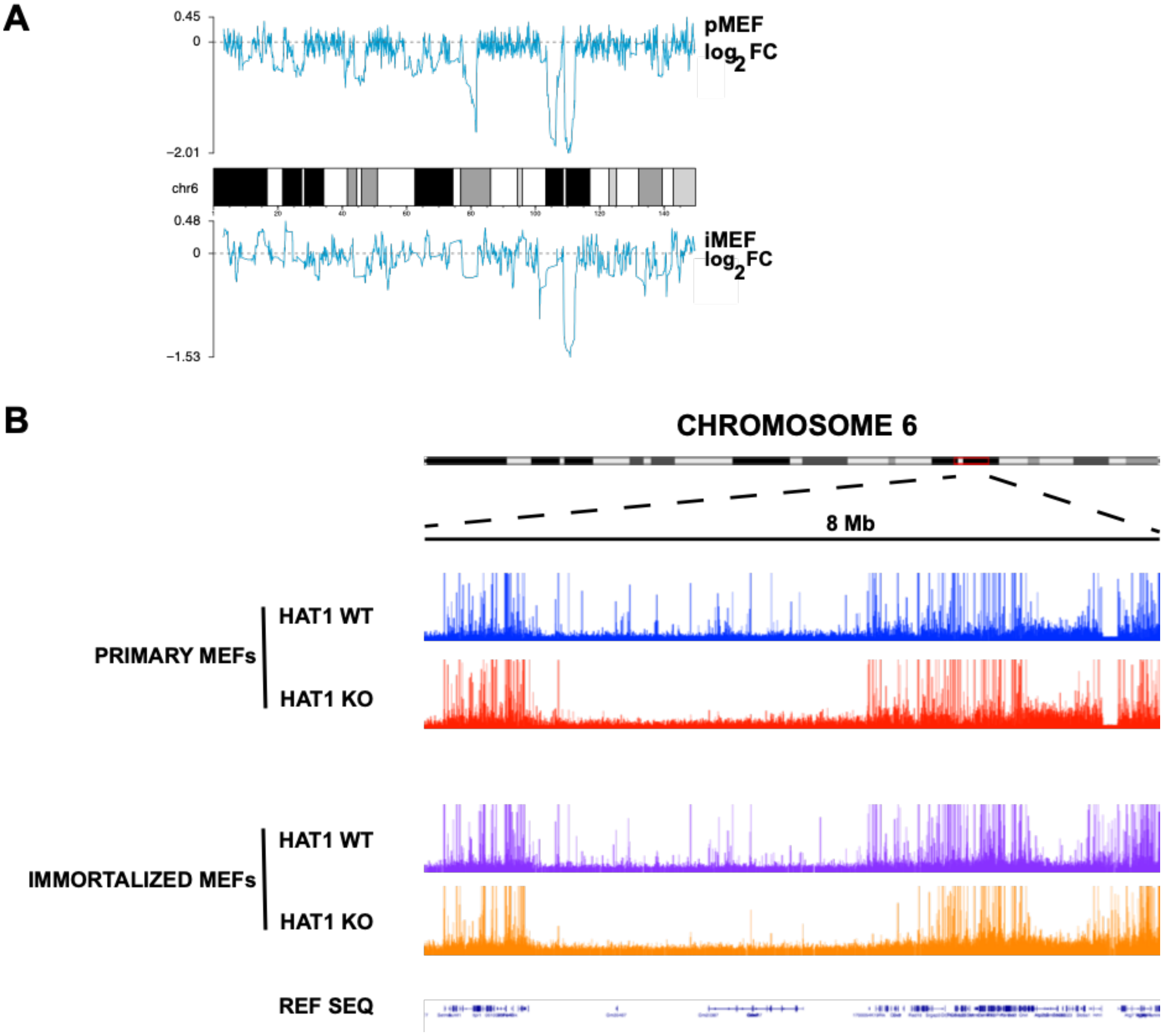

Supplementary Figure 2

A

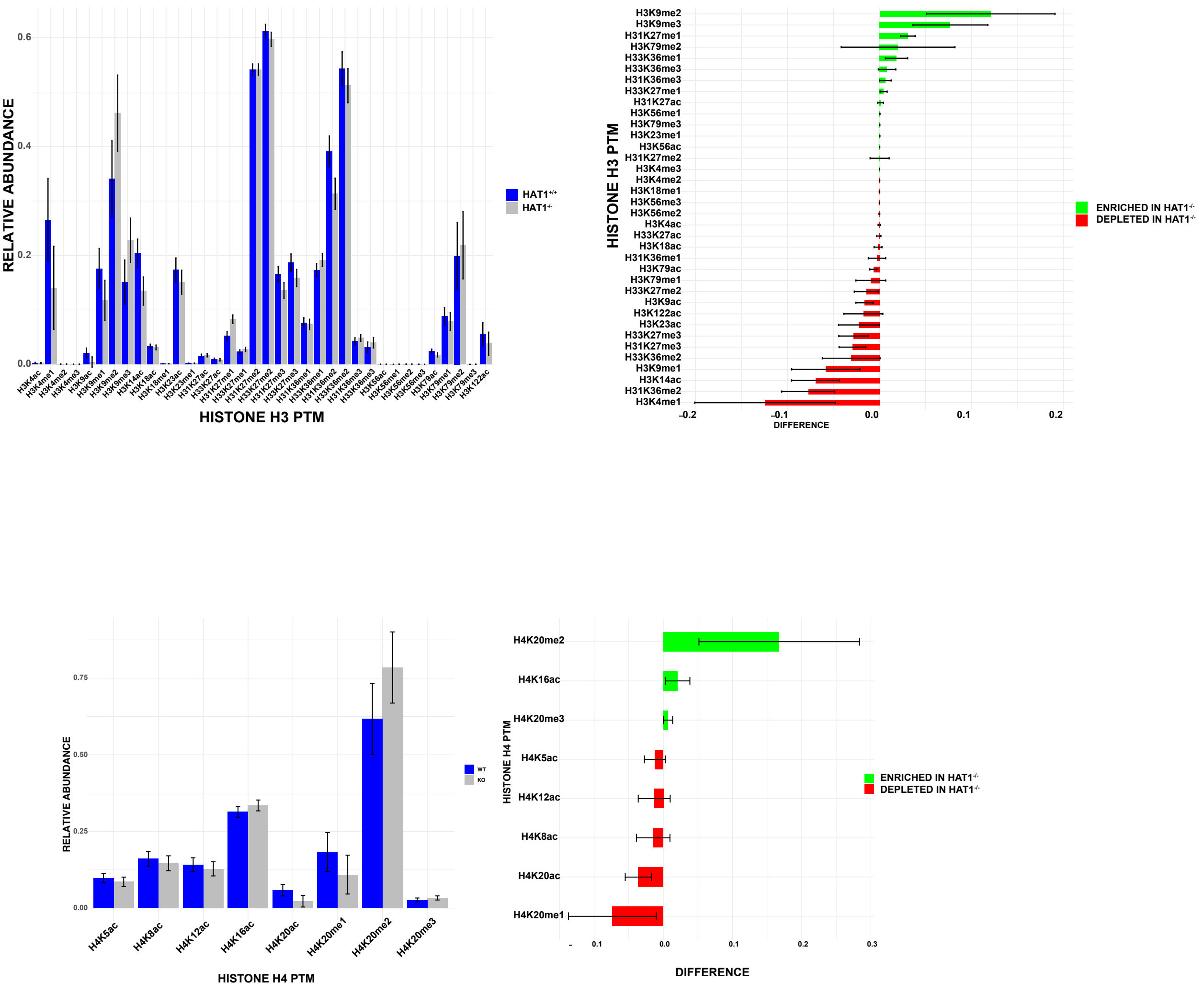

B

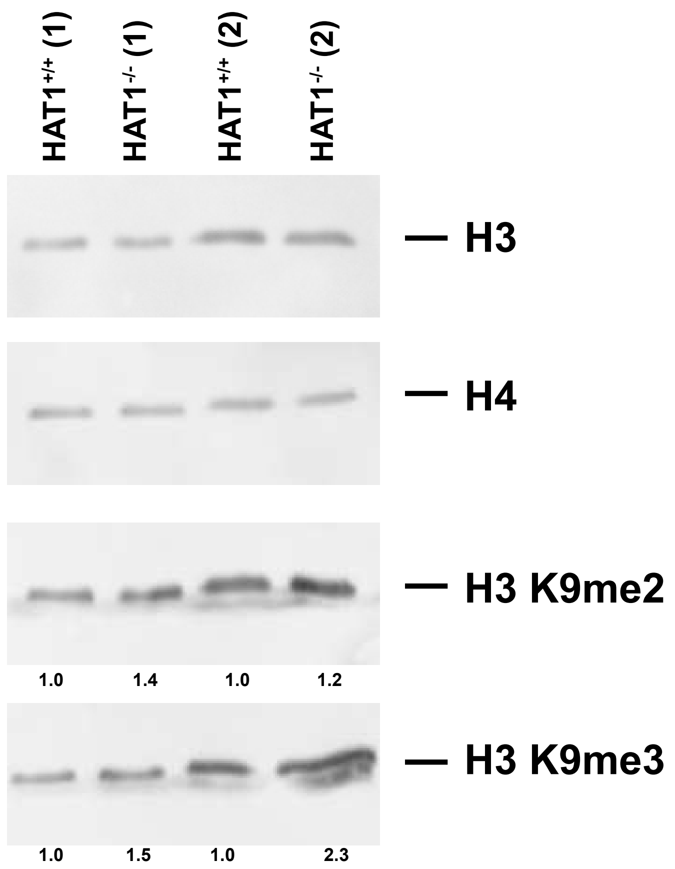

Supplementary Figure 3

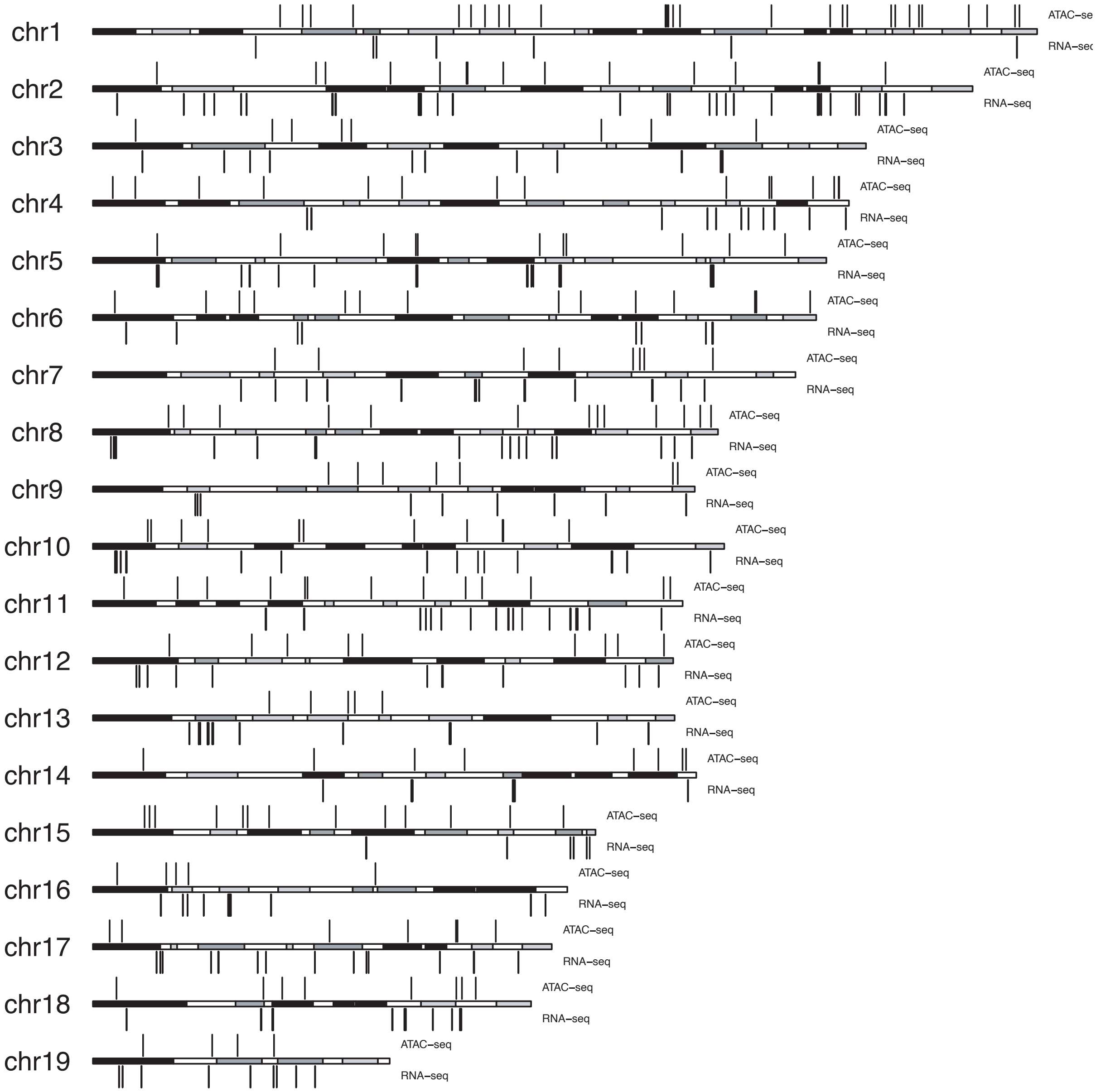

Supplementary Figure 4

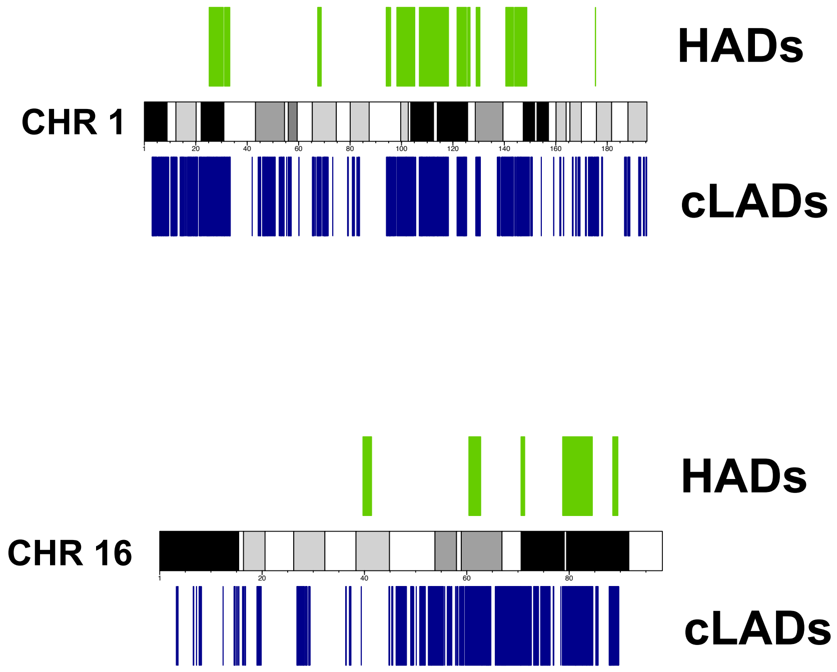

Supplementary Figure 5

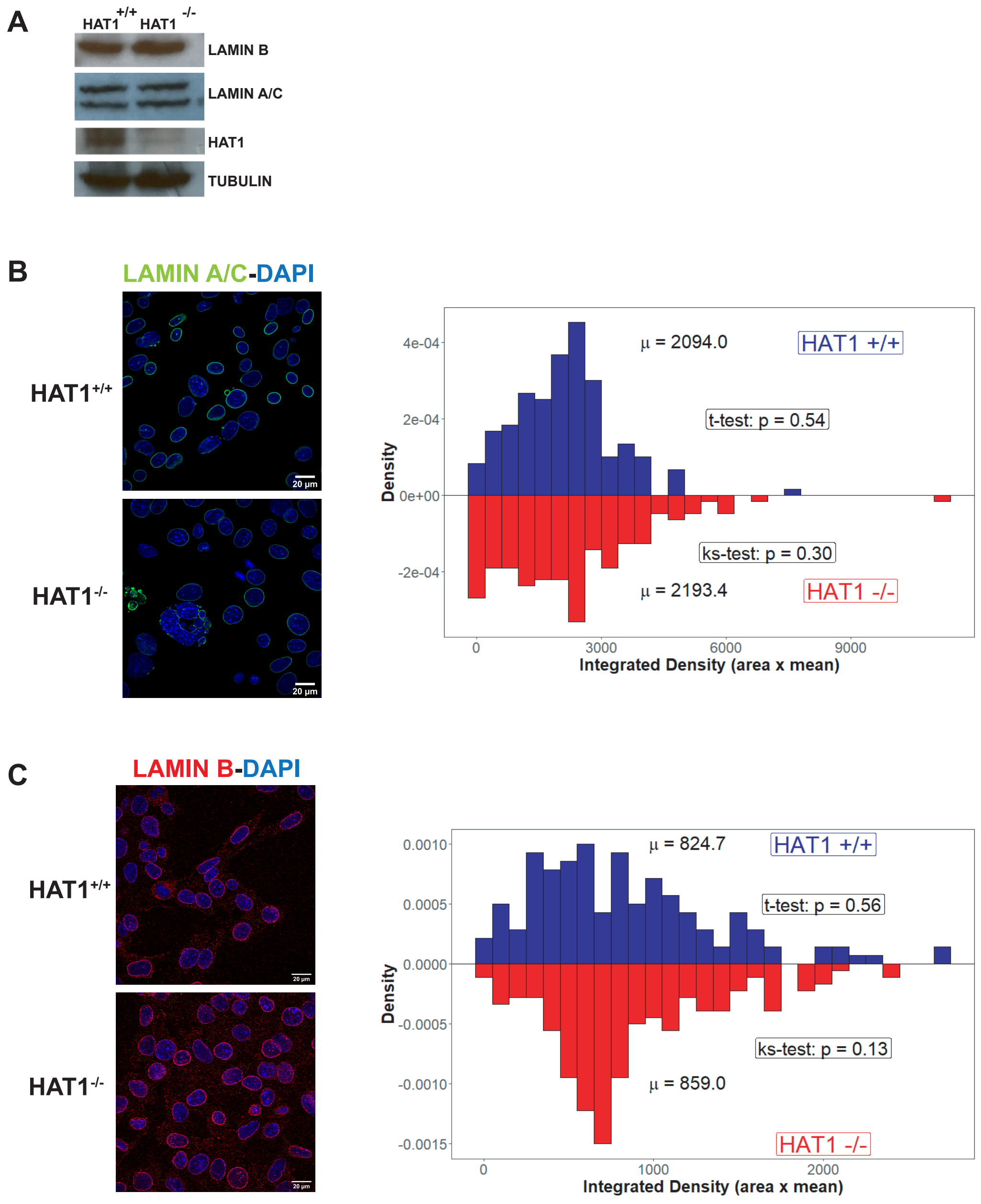

Supplementary Figure 6
